# Who Are Dentists?

**Published:** 1861-04

**Authors:** W. A. Pease


### WHO ARE DENTISTS?

BY W. A. PEASE.

IN a preliminary article a few lines were devoted to show that the first idea the community, in general, had of a dentist was, that he was a man who extracted teeth and made artificial ones—that he was a mechanic. This idea, which was true at the time it was formed, and is still true and applicable to many who call themselves dentists, does very great injustice to dentists,—men who have devoted their time and energies to the preservation of the natural teeth; and it, consequently, works an injury to them and a still greater injury to the community, for people are thereby often grossly deceived and lulled into neglect or a seeming unconsciousness of the decay going on in their own or their children's teeth; thinking (if they think at all) that dentists are essentially mechanics, and that they have but little control over the diseases of the teeth. It will be the aim of these papers to show the groundlessness of this opinion—to show that dentists can preserve the natural teeth, and that, consequently, the worst possible duty that can be required of them is to ask them to render a less valuable service, and make artificial ones; that where people have done their whole duty to themselves, their children and to dentists, artificial teeth should be as seldom required as artificial legs or glass eyes.

Commencing, then, in the order of arrangement as indicated by popular belief, this article will be devoted to the extraction of the teeth, to show where, from neglect or other cause, a tooth has become worthless, the necessity of the operation, independent of any pain the tooth may give, and that a judicious extraction of seeming valuable teeth is often an important part of conservative dentistry.

The causes that now induce dentists to extract the teeth are different from what they were a few years since. Then they were extracted because they ached, or because there was a gum-boil at the root of them; and they were seldom extracted for other reasons. Now these causes, of themselves, are not considered sufficient; there must be other and paramount reasons in addition to them to induce a dentist to extract a tooth; or, if he does extract it, it is because the patient is either too poor, or he refuses to pay the fee necessary for treating and saving the tooth; in which case, it is much better for the patient that the tooth should be removed than that it remain in the mouth. The effect of carious teeth on the mouth, by affording a lodgment for food to undergo fermentation, is to vitiate the secretions, render them acrid and acid, which act as a solvent of other teeth. Cases are seldom seen where caries has appeared between the teeth and attacked one tooth but that, immediately opposite, on the adjoining tooth, there is found more or less of a cavity. If then, under these circumstances, the tooth having the larger cavity is extracted, the decay in the remaining tooth often becomes stationary or dormant, or progresses more slowly than before. Nor are the deleterious influences of roots or decayed teeth confined to the destructive effects known to be exerted upon the healthy teeth. These are of minor importance, compared to the long list of nervous or debilitating diseases; dyspepsia, or loss of tone of the stomach, and irritability, if not disease of the lungs, for which the sufferer sometimes undergoes a long course of general or constitutional treatment, with little benefit, simply because the local cause is ignored or overlooked.

After the destruction or the spontaneous death of the nerve, if it is not removed from the tooth, or the tooth is not treated to place it in a healthy condition, there are few systems sufficiently healthy or robust to resist the irritability arising from that dead nerve, and other causes, not readily explained to the general reader. The result is, the tooth becomes sore; it is longer than the others; it is painful, and there is a swollen face, or what is popularly called a gum-boil. After the pus has discharged, which is either through the gum, or it insensibly escapes through the canal in the root, causing an unpleasant taste in the mouth, the disease becomes chronic, that is, lasting, and there is a constant oozing or discharge of pus into the mouth, which is insensible, and consequently swallowed, to the injury of the stomach and to vitiate the blood. Hence it is very bad policy to destroy the nerve in a tooth, unless the tooth is to be immediately plugged, or else, for some temporary purpose, such as in sickness, with a view of having it extracted at the first favorable opportunity before ulceration takes place. Independent of these considerations, it is a well known fact that the depressing effect of a small discharge of pus is equivalent to the loss of a considerable quantity of blood; and when this discharge is going on silently, but constantly, for days and years in the mouths of thousands of people, especially of females, is it surprising that the community is full of nervous, irritable, emaciated people ; constantly complaining, having no particular disease or pain beyond a general debility, or an occasional neuralgic twinge ; who are commiserated by their friends and called fidgety by the doctors? These people are in effect daily bled.

Thanks to dentists, the effects of the diseases of the teeth on the system are now beginning to be understood ; and a long list of ailments that have generally been intractable are now brought to a summary end, either by curing the diseased tooth or removing it from the mouth.

Thus, having shown some of the reasons why certain diseased teeth and all roots should be removed from the mouth, I shall now proceed to show why other, and at times healthy or seemingly valuable teeth should be extracted. Paradoxical as it may seem, the extraction of certain teeth is, occasionally, an important part of conservative dentistry; and judiciously exercised, it exerts as controlling an influence on the health of the mouth, the beauty and symmetry of the features and the preservation of the remaining teeth, as any one thing at the disposal of the dentist. While it is, undoubtedly, true that thousands of teeth have been and are still daily extracted, unnecessarily and for no adequate cause, it is also true that teeth are occasionally filled that it would have been much better for the patient to have extracted. As the chief object of dentistry is to preserve the teeth, dentists must keep steadily before their eyes, in all operations, the means for the attainment of that end. They should never be diverted from this duty by the position or solicitation of the patient, and however gratifying it may be to them, having an able and liberal patron, to show their control over disease and their ability to form and build up a solid and durable plug, if, from the nature of the case, the tooth may soon break away from the plug, the operation then becomes a glittering professional curiosity, unworthy the expenditure of the skill and the time it has consumed of the patient and operator. It is not to be understood from this, that teeth are not to be filled, when, from the nature of the case, the tooth must be ephemeral and transitory; but that such operations should not be performed when the extraction of the tooth would conduce, materially, to the health and permanence of the remaining teeth. Thus, a tooth that it would be proper and the duty of a dentist to plug for a patient of forty years of age, it might be his duty to extract, and it would verge on mal-practice to plug it, for a person of sixteen. In the one case, the condition of the system is stationary; the person has no more teeth to cut; his osseous system has become consolidated; the teeth are fixed and immovable, and they have attained their maximum density; they are less susceptible of disease, and when attacked by caries, it progresses more slowly than in early life, and consequently, a plug is then more efficient; while, if the tooth were extracted, nothing could be gained by a re-arrangement of the teeth, and injury might follow. On the contrary, if the tooth was plugged for a person of sixteen, the plug, as a plug, might be a success, but the tooth, being friable, might crumble away, and the root would have to be extracted at an age when the person would derive little benefit from the operation. At the age of sixteen, or even twenty, the system is still plastic; the natural arrangement of the teeth may be easily changed; the wisdom teeth are yet to appear, or are about cutting, and if a tooth is extracted, the teeth back of it will come forward and nearly fill the place, so that the teeth will stand a little asunder, and can be more easily cleaned, and the wisdom teeth, having abundance of room, are healthy and better developed. All of these advantages would be measurably lost if the tooth was plugged, and it afterwards broke away and had to be extracted at twenty-five. Thus it will be seen that the operations of the dentist are relative and conditional, dependent upon a great variety of circumstances, and his success will be measured by his ability to discover these conditions, interpret their significance, and adapt his treatment to the peculiarities of the individual case.

